# Examination of a foot mounted IMU-based methodology for a running gait assessment

**DOI:** 10.3389/fspor.2022.956889

**Published:** 2022-09-06

**Authors:** Fraser Young, Rachel Mason, Conor Wall, Rosie Morris, Samuel Stuart, Alan Godfrey

**Affiliations:** ^1^Department of Computer and Information Sciences, Northumbria University, Newcastle upon Tyne, United Kingdom; ^2^Department of Sport, Exercise and Rehabilitation, Northumbria University, Newcastle upon Tyne, United Kingdom

**Keywords:** algorithm, gait, inertial measurement unit (IMU), running, wearable

## Abstract

Gait assessment is essential to understand injury prevention mechanisms during running, where high-impact forces can lead to a range of injuries in the lower extremities. Information regarding the running style to increase efficiency and/or selection of the correct running equipment, such as shoe type, can minimize the risk of injury, e.g., matching a runner's gait to a particular set of cushioning technologies found in modern shoes (neutral/support cushioning). Awareness of training or selection of the correct equipment requires an understanding of a runner's biomechanics, such as determining foot orientation when it strikes the ground. Previous work involved a low-cost approach with a foot-mounted inertial measurement unit (IMU) and an associated zero-crossing-based methodology to objectively understand a runner's biomechanics (in any setting) to learn about shoe selection. Here, an investigation of the previously presented ZC-based methodology is presented only to determine general validity for running gait assessment in a range of running abilities from novice (8 km/h) to experienced (16 km/h+). In comparison to Vicon 3D motion tracking data, the presented approach can extract pronation, foot strike location, and ground contact time with good [ICC_(2,1)_ > 0.750] to excellent [ICC_(2,1)_ > 0.900] agreement between 8–12 km/h runs. However, at higher speeds (14 km/h+), the ZC-based approach begins to deteriorate in performance, suggesting that other features and approaches may be more suitable for faster running and sprinting tasks.

## Introduction

Running has become one of the most popular sports, promoting health benefits through increased physical activity while remaining readily accessible to all (Shipway and Holloway, 2010). With the increased uptake of running and running-based exercise, the incidence of lower-extremity injuries associated with running, has risen e.g., Achilles tendinopathy and Plantar Fasciitis, (Dempster et al., 2021). Injuries, if not treated, can exacerbate in beginner and novice runners (Linton and Valentin, 2018), with a higher injury rate linked to self-devised, informal training plans compared to well-informed approaches, i.e., novice runners may not access or are unaware of information leading to efficient, safe running practices to minimize injury. Prominently, it has been shown that a high incidence of injuries is due to impact-load management issues where a better understanding of the biomechanical properties within each running style plays a key role in understanding the type of injury. For example, rear-foot (heel) strikers are significantly more likely to incur an injury than those who do forefoot running (Daoud et al., 2012). More specifically, over-pronation during running can lead to medial tibial stress and plantar fasciitis (Rolf, 1995; Hintermann and Nigg, 1998).

Selection of the correct running equipment, such as shoe type, has been shown to minimize injury risk by optimizing load distribution through the use of various cushioning technologies. For example, a support shoe will include anti-pronation cushioning to minimize the roll of the foot on impact (Jafarnezhadgero et al., 2019). However, challenges arise when selecting the correct running shoe. Typically, a runner is manually assessed by an individual who (i) visually assesses the fall of the foot during walking or running overground or on a treadmill and/or (ii) observes the wear pattern of previous running shoes to understand pronation severity and foot strike location (Higginson, 2009). However, visual assessments are intrinsically flawed through a lack of subjectivity and reliability (Higginson, 2009). In particular, it has been shown that while the visual assessment of foot strike pattern is highly accurate, visually assessing pronation is unreliable between assessors, with agreements between 42 and 56% (Meyer et al., 2018). As such, the use of technology is essential for accurate instrumentation of running gait.

Three-dimensional (3D) motion capture video-based systems are generally considered the reference/gold standard in gait assessment, consistently demonstrating validity and reproducibility in a range of applications (Baskwill et al., 2017; Albert et al., 2020; Jakob et al., 2021). However, the use of a 3D motion tracking system demonstrates obvious pragmatic issues through high costs, an intrusive nature (i.e., users must be fitted with a range of anatomical markers), as well as the need for technical expertise (Schlagenhauf et al., 2018; Sharma et al., 2019), limiting the use of technology in low-resource, real-world settings. As such, wearable inertial measurement units (IMU) have seen a recent usage uptake in running gait assessment by providing a low-cost apparatus capable of detecting intricate running gait outcomes (Young et al., 2020; Benson et al., 2022). Typically, IMUs contain a combination of inertial accelerometer and gyroscope sensors to provide an understanding of acceleration and rotation (Ahmad et al., 2013). IMUs can measure a wide range of running biomechanics, including gait phase estimation (Sui et al., 2020; Young et al., 2021), impact analysis (Tan et al., 2020), flexion angles (Cooper et al., 2009; Nagahara et al., 2020), foot orientation (Falbriard et al., 2020), and asymmetry measures (Ueberschär et al., 2019; Benson et al., 2022). Crucially, their use enables reproducible, objective gait outcomes that can enable standardization within the domain, especially in opposition to traditional visual assessments (Higginson, 2009; Chew et al., 2018; Benson et al., 2022). Furthermore, with a small form factor and relatively low cost, IMUs can measure beyond the lab (Strohrmann et al., 2011; Benson et al., 2022), which can help understand running gait of varying lengths in a variety of environments, from short capture sessions (e.g., sprinting Schmidt et al., 2016) under observation in low-resource settings to prolonged periods over the ground, e.g., marathons (Meyer et al., 2021).

IMUs are typically reliant on algorithms to extract useful gait features from inertial signals. Algorithms can generally be described as software-based methodologies, translating raw (sample level) data into meaningful and quantifiable gait outcomes. To date, a plethora of IMU-based algorithms have been developed for running gait assessments (Mason et al., 2022). Typically, algorithms rely upon the identification of initial contact events from the inertial data to segment the gait cycle for specific phases of analysis (Gujarathi and Bhole, 2019; Young et al., 2021). One common approach is the zero-crossing (ZC) technique (Mason et al., 2022), which is particularly useful when used in conjunction with other inertial feature extraction methods such as gradient maxima (i.e., peak detection)—often used to extract peaks in corresponding inertial signals (Alahakone et al., 2010; Norris et al., 2014). In particular, bouts of running gait naturally exhibit higher acceleration during impact, creating easily identifiable peaks within acceleration signals, justifying the use of a ZC gradient maxima algorithm to segment gait. Consequently, using an accelerometer can inform gyroscope-based outcomes (e.g., foot roll and impact location). This benefit of that multi-modal sensing strategy is particularly evident when IMU devices are placed on the lower extremities (e.g., feet), increasing the sensitivity to ground-impact biomechanical-related inertial signal features (Panebianco et al., 2018), which has been demonstrated in running gait (Alahakone et al., 2010; Young et al., 2020).

Furthermore, ZC has pragmatic utility in comparison to recent approaches such as artificial intelligence and machine learning within the wider gait assessment field, which, despite their ability to provide comprehensive gait outcomes (Zhang et al., 2019; Xu et al., 2022), are both computationally intensive (Khera and Kumar, 2020) and require complex logistics such as setup, training, and hosting. Conversely, ZC methods require low computational power for more immediate deployment for running gait assessment tasks (Hölzke et al., 2020), as the technique is more readily deployable (i.e., does not require the building of datasets and complex implementation), indicating suitability for low-resource deployment, e.g., on a tablet or smartphone without cloud connectivity. Previous work (Young et al., 2020, 2021) developed a low-power IMU ZC methodology with a foot-mounted IMU to assess biomechanical properties such as foot strike location, pronation, and ground contact time to recommend shoe type while participants ran at a single set pace (8 km/h). Nevertheless, the validity of the ZC approach has not been investigated comprehensively for general use in running analysis. Here, we conduct a thorough investigation of the fundamental ZC methodology for foot strike identification and pronation ground contact time at varying speeds, cross-referenced with a 3D-motion capture system and slow-motion video reference streams. We hypothesize that the ZC is a useful approach for examining running gait outcomes across a range of speeds.

## Methods

### Participants

Ethical approval was granted by the Northumbria University Research Ethics Committee (Reference: 21603). All participants were provided with the necessary information before participating, and they gave verbal and written consent before performing treadmill-based testing in Northumbria University's Gait and Biomechanics Laboratory. One-third of the healthy participants (34.5 ± 9.67 years; 1.75 ± 0.3 m; 76.2 ± 4.1 kg; 20M:11F) were recruited from running clubs in the Northeast of England. Participants exhibited a range of running abilities from ≈30 min (amateur) to ≤ 20 min (experienced) for a 5 km pace. Inclusion criteria required that participants could run unassisted for short periods and must be under the age of 60 years. Participants were screened for running-related injury history, as well as any gait/mobility-affecting conditions (e.g., orthopedic and cardiovascular) that would adversely impact running ability. No participants reported any current running gait-affecting injuries or pre-existing conditions to warrant exclusion. Participants were provided with a standardized, neutral-cushioning running shoe (Saucony Guide Runner) for use during testing to minimize impact at higher speeds.

### Instrumentation: IMU

**Figure 1:**
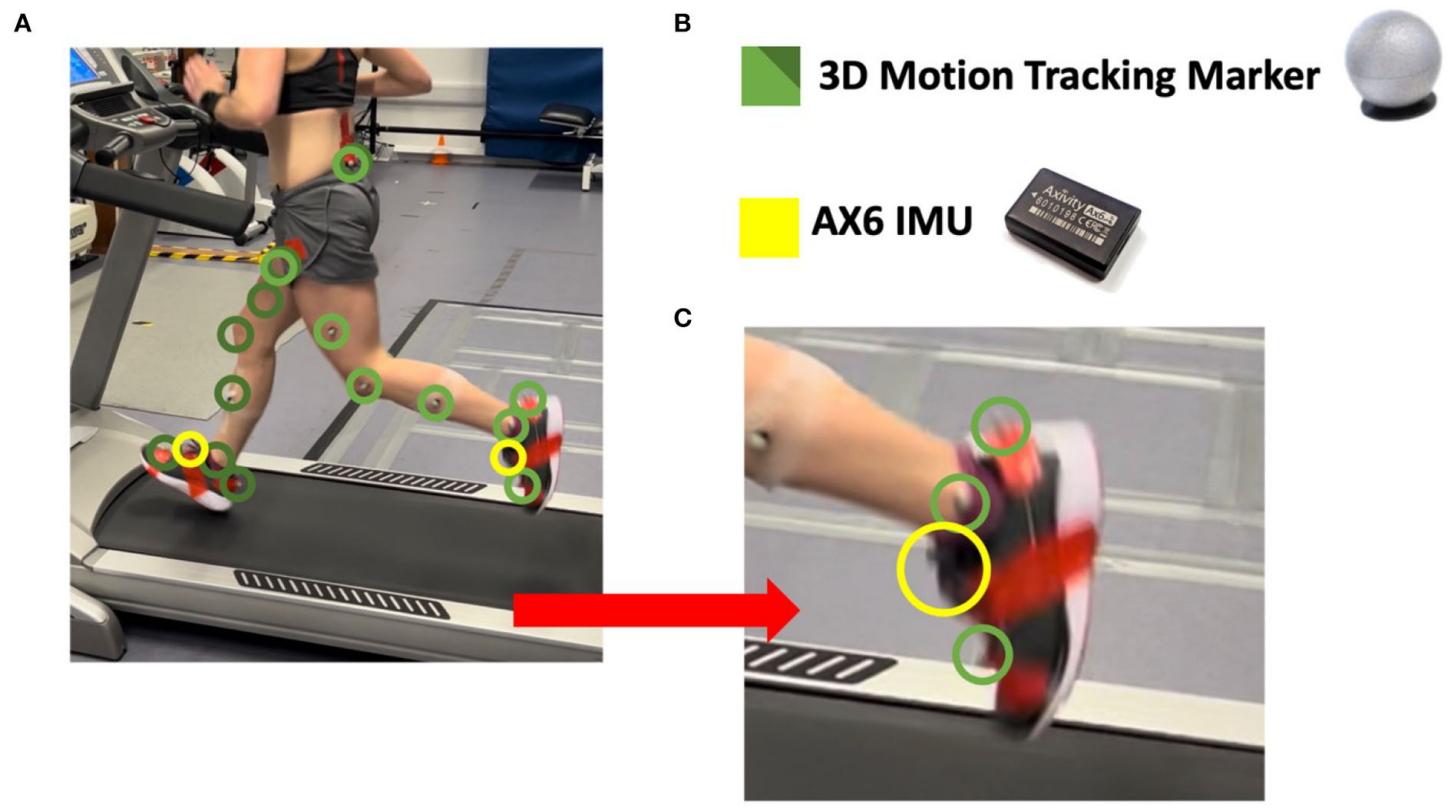
Example of a participant mounted with the full range of markers and sensors. **(A)** A macro view of a runner during testing, donning **(B)** 16 neo-reflective 3D motion tracking markers and 2 AX6 IMU devices. **(C)** Illustrates a zoomed view of the foot with the markers at the heel, ankle, and front-foot positions; with the AX6 at the talus joint of the foot.

All participants were fitted with two wearable IMUs (AX6, Axivity, UK, https://axivity.com/, tri-axial accelerometer, and tri-axial gyroscope, 23.0 × 32.5 × 8.9 mm, 11g) on the talus joint of each foot with medical tape (Figure 1). IMUs were programmed in Axivity's omGUI software suite, configured with ± 16 g accelerometer range, and 2,000 dps gyroscope range polling at 60 Hz. The location of the IMU on the talus is essential to reproducing the ZC methodology under investigation. Specifically, tracking the orientation of the talus provides an optimal representation of foot rotation throughout the running gait cycle (Hontas et al., 1986) to determine the foot strike pattern, pronation, and ground contact time.

### Instrumentation: Reference

For standard reference, a three-dimensional (3D) 14-camera motion tracking system (Vertex, Vicon, UK, www.vicon.com) was used. The 14 Vicon Vertex motion tracking cameras were distributed around a space of 9.8 × 7.9 × 3.2 m^3^, polling at 200 Hz to provide a high-resolution observation of the participant's running gait. Participants were fitted with 16 neo-reflective markers for use with the Vicon 3D motion tracking system in the following locations: (1) calcaneal tuberosity (heel), (2) lateral malleoli (ankle), (3) base of the second metatarsal (front-foot/toe), (4) lateral mid-shank, (5) lateral knee joint line, (6) mid-lateral thigh, (7) anterior superior iliac spine, and (8) posterior superior iliac spine (Figure 1).

### Data capture

Participants initially performed a static pose (arms to the side and feet shoulder-width apart) to calibrate the 3D motion tracking system. Subsequently, participants were prompted to walk for short periods within the 3D tracking environment, providing synchronized data between the 3D tracking, video, and IMU data streams. To ensure synchronization between Vicon and IMU data streams, digital timestamps were created consistently in software for both systems based upon the operating system clock in “milliseconds since epoch” format. Upon successful configuration, participants stood still on the treadmill to provide a baseline reading from IMU devices and to account for any local inclination error or misalignment during fitting. Participants were then asked to perform short, 1-min bouts of treadmill running at four set speeds (8, 10, 12, and 14 km/h) in line with guidelines in previous work (Young et al., 2020) and to ensure participants could successfully complete the tests despite their running ability. A period of 1-min was chosen, as it generally aligns with other similar studies in the field with data capture periods ranging from the 20 s (McGrath et al., 2012) to the 90 s (Bailey and Harle, 2014; Tan et al., 2020). Additionally, participants also ran at a speed comparable to their most recent outdoor 5 km pace (15.1 ± 0.8 km/h). If their self-selected pace was below or equal to pre-defined speeds, no self-selected pace was captured. All runs captured inertial, 3D motion, and video data (240 FPS side and rear perspectives). Tests were conducted twice after a short break (≈1 min) to provide multiple running bouts for participants at each pace. As such, a total of 148 running bouts were assessed during this study.

### Data labeling

**Figure 2:**
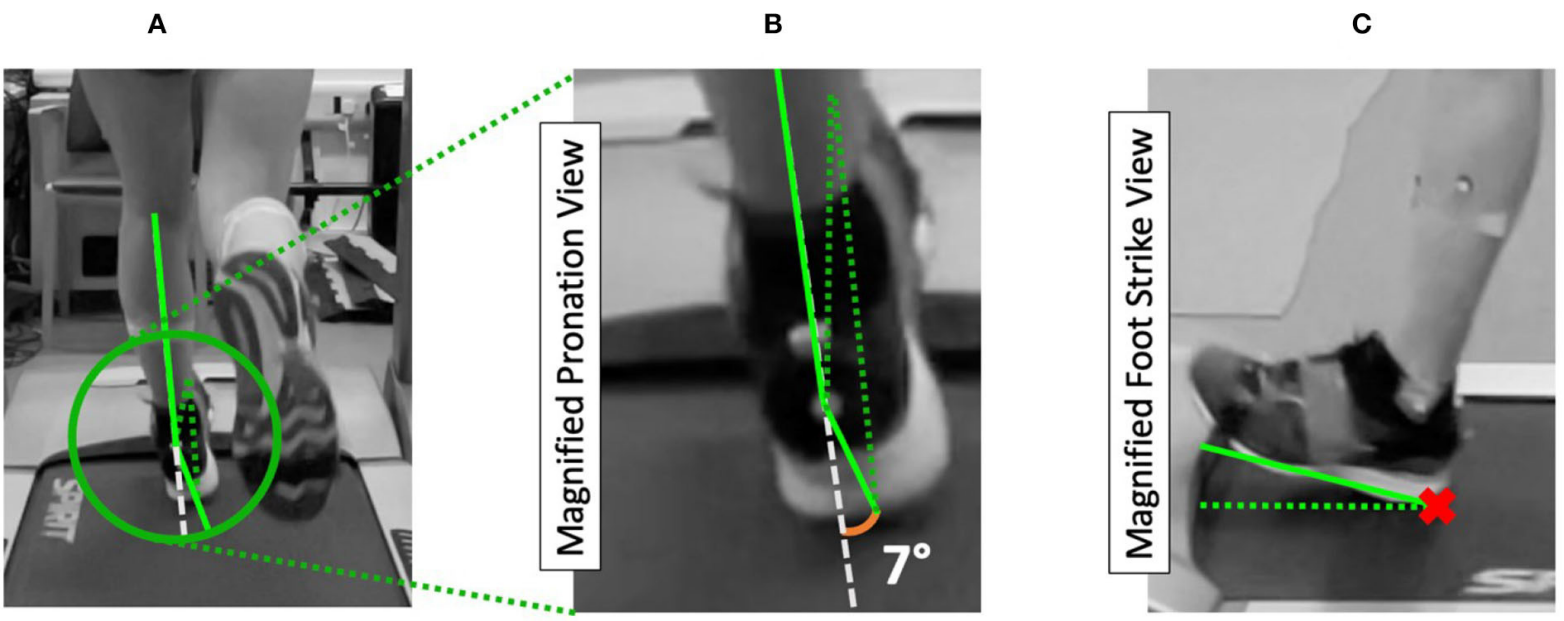
Illustrated the view of the labeling process of pronation and foot strike between three neo-reflective markers located at the ankle, heel, and frontfoot positions. The pronation angle is derived based on the angle of the leg in comparison to the angle of the foot, **(A,B)**. Foot strike **(C)** is determined as the first point that made contact with the ground (red x): heel, mid, or forefoot.

Foot strike location, pronation severity, and ground contact time were manually labeled by-hand through observation of 3D motion tracking data and slow motion video reference streams in accordance with labels of the previous studies (Young et al., 2020, 2021) by a team of trained researchers (sports science biomechanics) such that each foot may exhibit: neutral, slight or pronated roll of the foot; or heel, mid or fore foot strike location (Figure 2). Labels are generated by observing skeletal output generated by the Vicon system in cross-reference with slow-motion video streams such that pronation is the angle between the heel, ankle, and leg angle (Figure 2B), and foot strike location denotes the impact location of the foot (Figure 2C). A runner is considered pronated should they exhibit 5° or greater foot roll during initial contact, in line with previously outset guidelines (Young et al., 2020). Due to different sampling resolutions between 3D motion capture (200 Hz) and IMU signals (60 Hz), ground contact time is measured and labeled with respect to milliseconds to standardize the measurements. For example, ground contact time from 3D motion capture could output 38 Hz, whereas the IMU could output 11 Hz. Consequently, each method is resampled to 190 ms and 183 ms for 3D motion tracking and IMU data, respectively. Ground contact time is measured as the time elapsed (ms) between initial contact (foot first makes contact with the ground) and final/terminal contact (foot last leaves the ground). Of the 148 running bouts observed, a total of 9,327 strides (mean steps per test = 57.2 ± 4.09) were extracted, labeled, and assessed as part of the study.

### Data processing and the algorithm

Data handling and processing have been described previously (Young et al., 2020, 2021). In brief, acceleration and rotation data are extracted and analyzed in a Jupyter notebook Python 3.7 environment for the execution of the algorithm. Data were prepared/filtered by a Butterworth band-pass filter performing at 60 Hz with a sampling frequency of 3 Hz, and a cut-off frequency of 5 Hz is applied to the vertical acceleration plane and vertical/horizontal rotational velocity to account for signal noise.

### IMU algorithm methodology

**Figure 3:**
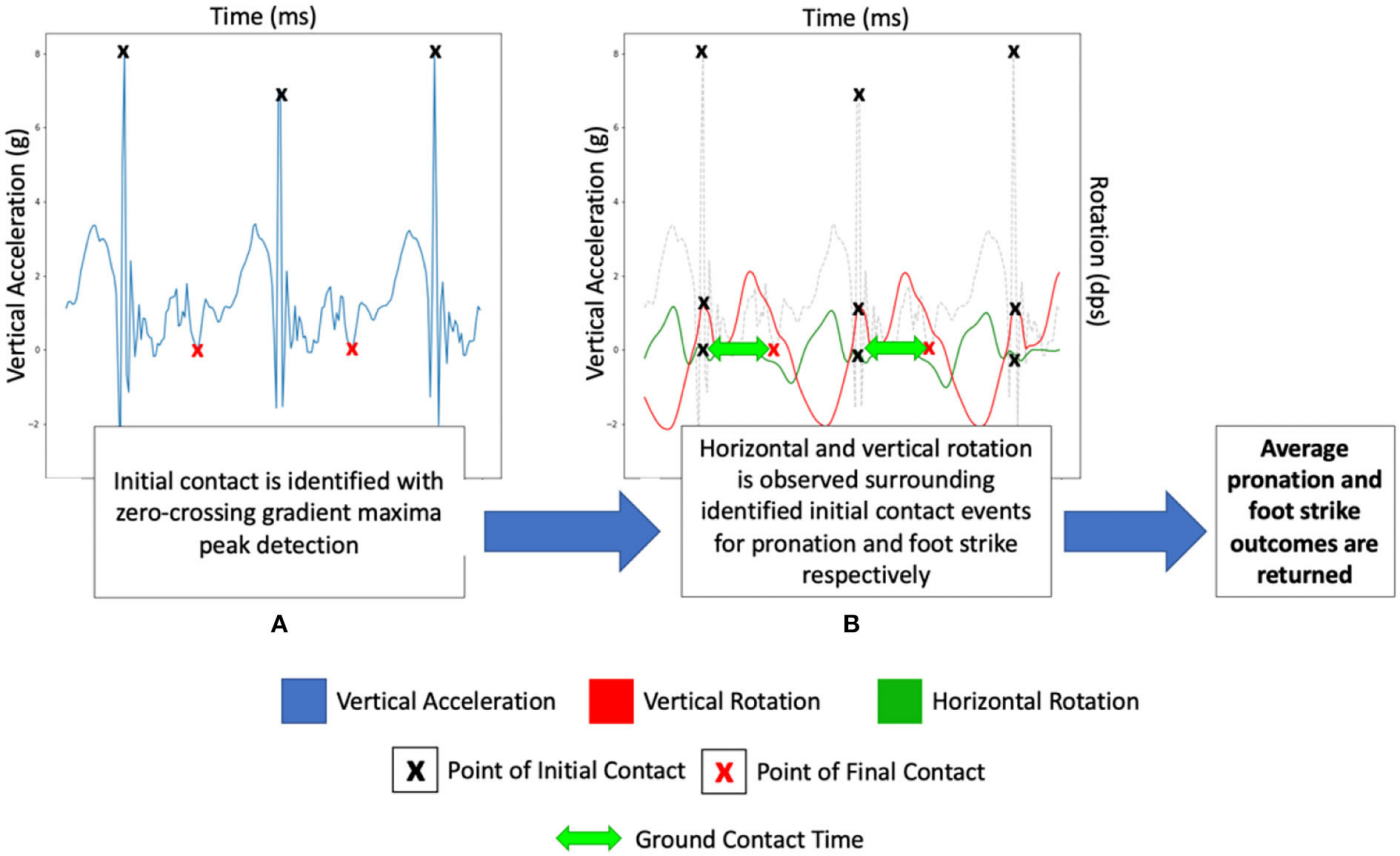
Data illustration of the evaluated algorithm at the key stages of execution **(A)** initial/final contact identification and **(B)** gait feature extraction at the point of contact.

The method analyses tri-axial accelerometer and tri-axial gyroscope signals in tandem during running to quantify foot strike location, pronation severity, and ground contact time. The method relies on the identification of initial contact from ZC to inform gait feature extraction surrounding impact. A short breakdown of the algorithm is presented below:

(a) Initial contact identification (Figure 3A): A ZC gradient maxima algorithm is deployed for detecting the peaks in the vertical acceleration plane; it is deployed for initial contact identified by observing significant gradient changes. Operating within a dynamic threshold based upon the signal maxima, the ZC gradient maxima algorithm effectively identifies initial contact peaks in vertical acceleration.
(b) The rotational velocity of the foot in vertical and horizontal planes is observed around identified points of initial contact (Figure 3B). An average is taken of each feature, providing a final output of pronation severity and foot strike location.
(c) Final contact identification and ground contact time estimation: The same ZC gradient maxima algorithm is used to identify an inverse peak in the acceleration signal within a 500 ms region of interest following an identified initial contact event. Ground contact time is consequently calculated as the time between an initial contact event and the final contact event.

### Statistical analysis

The examination of the performance of the proposed algorithms and their respective videos was conducted in SPSS v27. The Shapiro–Wilks tests indicated a normal distribution of all data (p < 0.05). Consequently, intra-class correlation [ICC_(2,1)_] models examined absolute agreement between the IMU algorithm and 3D reference/slow motion video streams. A predefined ICC performance scale was deployed (Koo and Li, 2016), defined as poor (< 0.500), moderate (0.500–0.750), good (0.750–0.900), or excellent (> 0.900). The mean errors were calculated between the algorithm and 3D motion data for descriptive purposes and are observed as an accuracy metric in ground contact time. Furthermore, the Bland Altman plot (Bland and Altman, 1986) and the box plot were used to visually assess the agreement between ground truth and algorithm outcomes for ground contact time.

## Results

Of the 31 participants, no data loss or dropout was experienced during treadmill running sessions. Upon preliminary observation of the quantified algorithm outcomes, no significant outliers were identified. A total of 148 running bouts containing 9,327 strides were analyzed as part of the study.

### Initial contact

Intraclass correlation performance degrades at higher speeds in identifying points of initial contact, demonstrating excellent agreement between 8–10 km/h [ICC_(2,1)_ > 0.900) and good agreement (ICC_(2,1)_ 0.750) at 14 km/h and higher self-selected paces; see Table 1. The ZC gradient approach to initial contact identification tends to overestimate the number of initial contact events, especially at higher speeds.

**Table 1:** Initial contact identification performance in comparison to reference labels from 3D-tracking data at differing speeds.

	**Reference**	**Algorithm output**	
	**Average no. of steps**	**Average no. of steps**	**ICC (2,1)**
8 km/h	54	55	0.963
10 km/h	56	56	0.981
12 km/h	56	57	0.945
14 km/h	59	65	0.821
Self-selected Pace	60	72	0.783

The average number of steps denotes the mean steps per participant per foot across the range of datasets.

### Foot strike location and pronation severity

Intraclass correlations demonstrate excellent agreement between the algorithm and reference streams for foot strike identification (ICC_(2,1)_ > 0.900), particularly demonstrating robustness at the full range of speeds with low error rates throughout; see Table 2. Between 8–12 km/h, the pronation identification algorithm demonstrates good agreement (ICC_(2,1)_ > 0.750) but begins to depreciate at 14 km/h + with moderate (ICC_(2,1)_ > 0.500) agreement.

**Table 2:** Gait feature extraction performance in agreement with reference labels from 3D-tracking data at differing speeds.

	**8 km/h**		**10 km/h**		**12 km/h**		**14 km/h**		**Self-selected pace**	
	**Pronation**	**Foot strike**	**Pronation**	**Foot strike**	**Pronation**	**Foot strike**	**Pronation**	**Foot strike**	**Pronation**	**Foot strike**
**Mean error**	0.184	0.131	0.184	0.105	0.342	0.112	0.382	0.029	0.312	0.025
**ICC**_**(2,1)**_	0.867	0.918	0.833	0.915	0.779	0.915	0.687	0.987	0.712	0.989

### Ground contact time

**Figure 4:**
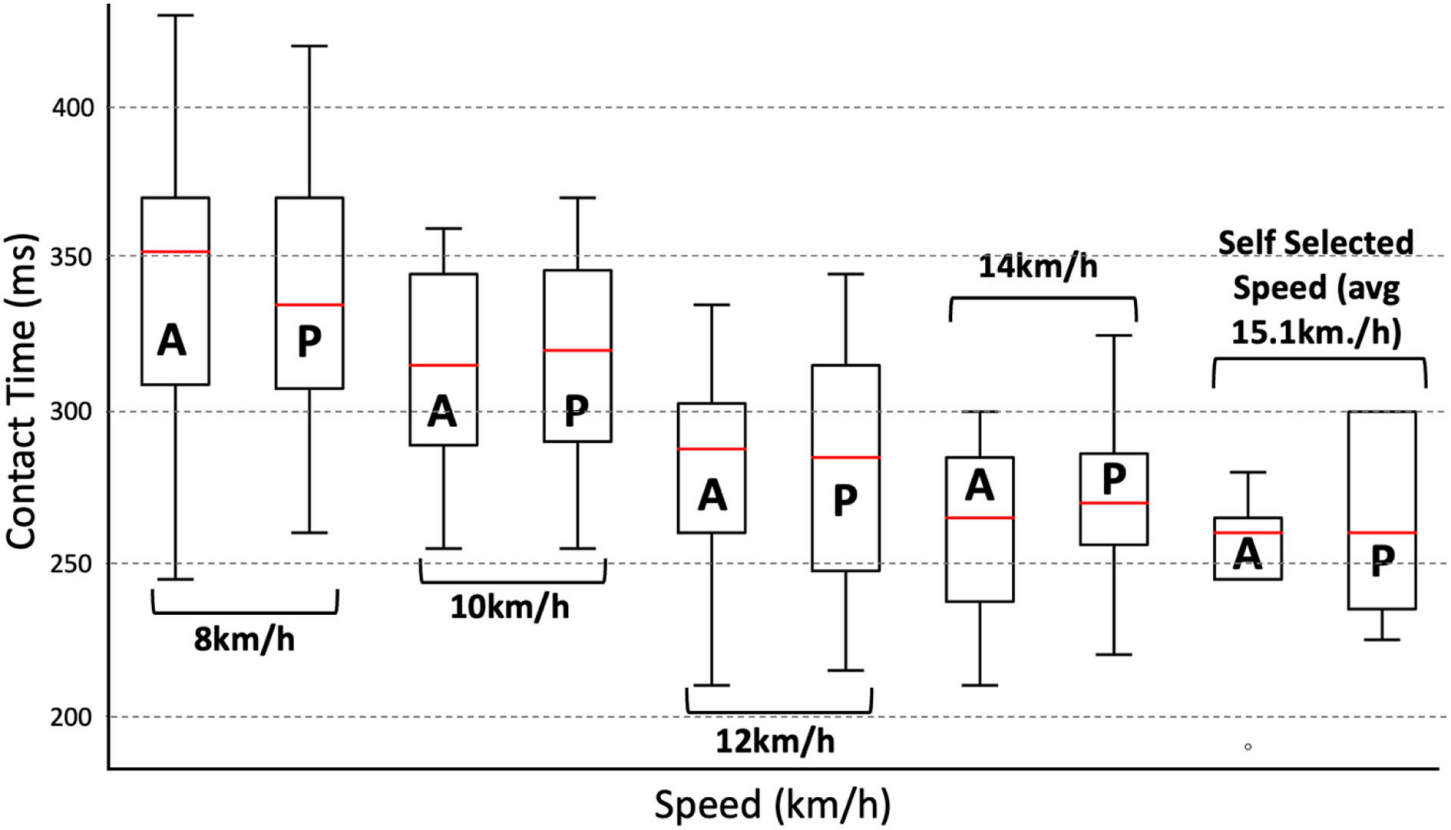
Box plots illustrating the performance of the contact time algorithm at 8, 10, 12, 14 km/h, and a self-selected pace. **A** refers to actual (labeled) contact time, and **P** refers to predicted (ZC algorithm) contact time.

The ground contact time identification approach demonstrates low mean errors at 8, 10, and 12 km/h (9 −17 ms) when compared to 3D motion tracking labels; see Table 3. In a similar vein, observing the median and upper/lower range at lower speeds demonstrates an ability to estimate the ground contact time of varying lengths (Figure 4). Conversely, the mean error rate is slightly higher (21 ms−27 ms) at the higher speeds (14 km/h +) while additionally demonstrating a wider deviance from the median and upper/lower range in comparison to labeled data (Figure 4).

**Table 3:** Performance of ground contact time extraction layer in comparison with labels from 3D tracking data between 8 km/h and a self-selected speed (avg = 15.1 k m/h).

**Speed (km/h)**	**8**	**10**	**12**	**14**	**Self-selected**
Mean algorithm output (ms)	335.5 ± 44.78	317.5 ± 31.52	282.5 ± 35.06	272 ± 27.86	272 ± 28.83
Mean labeled data (ms)	344.5 ± 46.55	316.5 ± 31.64	279.5 ± 33.06	261.5 ± 28.61	245.5 ± 29.95
Mean Error (Hz)	3.58	2.15	2.93	4.21	5.48
Mean Error (ms)	9	11	15	21	27
Mean Error (%)	2.65	0.32	1.07	3.94	10.24

Results are observed in milliseconds to standardize measurements between reference (Vicon; 200 Hz) and IMU (Axivity; 60 Hz) output. For illustrative purposes, the mean error is shown in both Hz and seconds and refers to the average error between the algorithm and labeled data.

## Discussion

Understanding running gait is crucial in injury prevention, particularly when quantifying pragmatic biomechanical properties. This can reduce impact-related or strain injuries commonly associated with over-pronation (Daoud et al., 2012). The proposed work investigates and evaluates the performance of a ZC methodology at different running speeds to assess suitability to quantify the foot strike location, pronation, and ground contact time. It was found that the ZC method had reduced agreement when compared to a standard reference at higher running speeds, suggesting its use for running analysis may be suitable for amateur runners only (i.e., those with a 5 km time > 20 min) compared to elite athletes and their gait assessment at higher speeds.

### Performance of zero-crossing methodology

In line with studies utilizing a ZC gradient approach for gait cycle segmentation (Bastas et al., 2018; Han et al., 2019), our IC identification algorithm demonstrated excellent absolute agreement across the lower range of speeds (ICC_(2,1)_ > 0.9) and good agreement (ICC_(2,1)_ > 0.75) at higher speeds by identifying peaks in vertical acceleration above a dynamic threshold. The ZC approach to initial contact identification demonstrates a mean error of between 9 and 27 ms between the algorithm and labeled output dependent upon speed; see Table 3.

**Figure 5:**
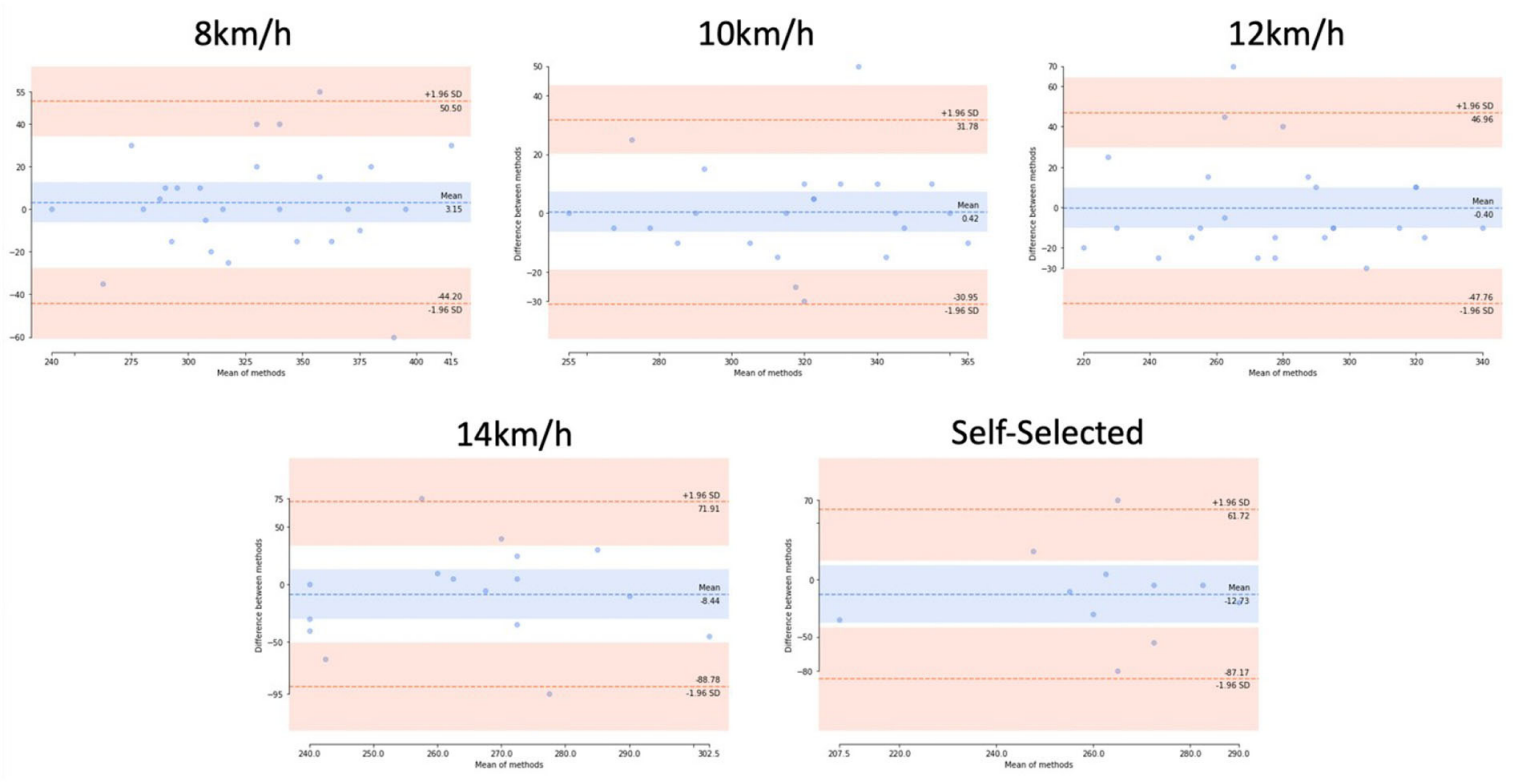
Bland-Altman plots of the ground contact time between ground truth and algorithm output. Blue and orange lines denote mean ± STD of the error.

**Figure 6:**
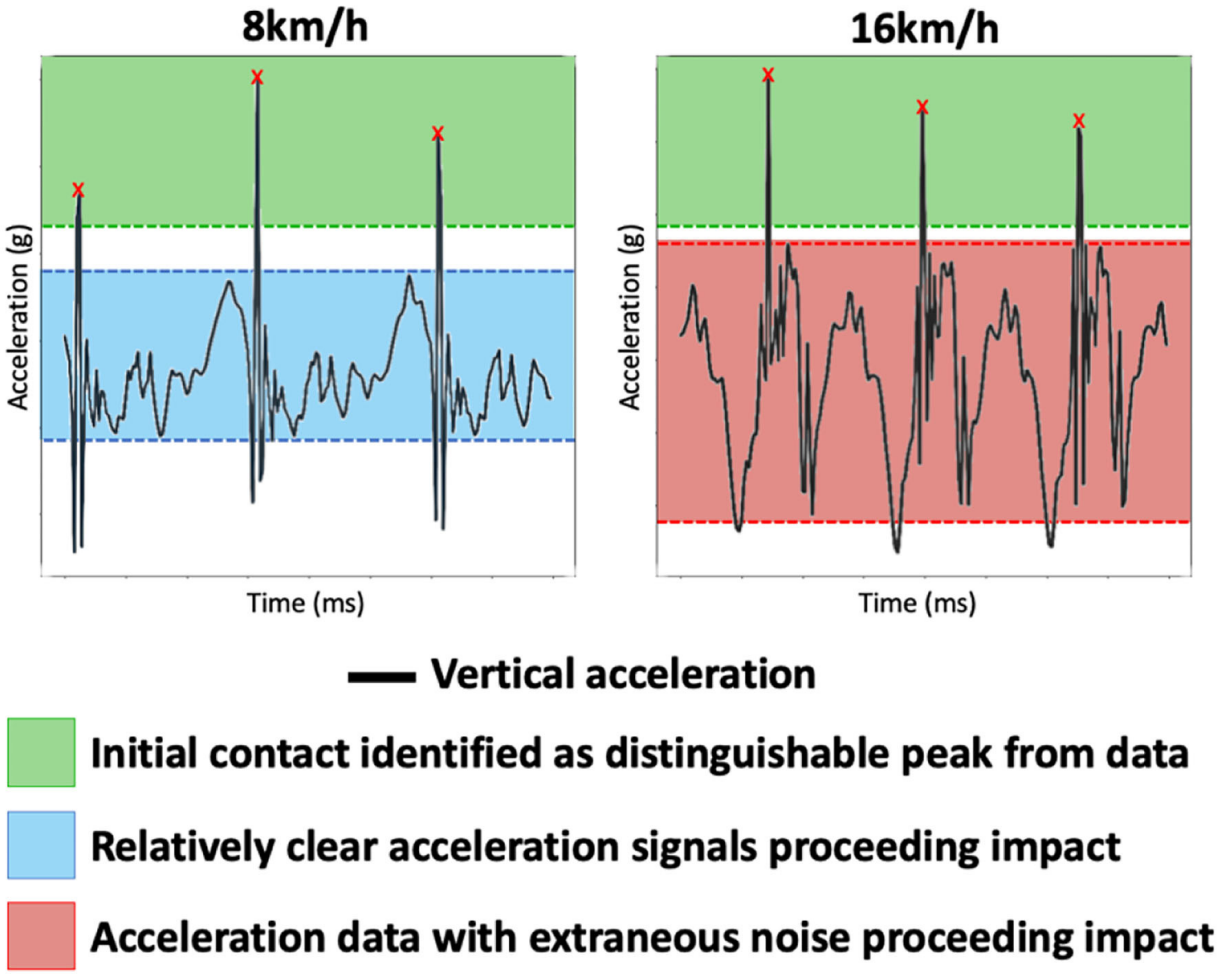
Comparison of a runner's vertical acceleration data at two different speeds, 8 km/h and 16 km/h. As observed, within higher speeds, a considerable increase in noise is noticed after an initial contact event. Additionally, the signals have obvious differences between speeds due to (i) noise and (ii) potential of changing gait with respect to speed (e.g., fore strike at 8 km/h and heel strike at 16 km/h). Consequently, extracting gait features around a point of impact could be significantly impacted at higher speeds.

By observing the Bland Altman plots of the initial contact identification approach at higher speeds (Figure 5), (14 km/h+), we could observe that it is evident that the approach successfully identifies labeled initial contact events, likely due to higher impact forces exerting significant vertical acceleration that gradient analysis can easily identify. However, false positives are occasionally encountered at higher speeds where extraneous noise is often present (Supplementary material, signal-to-noise analysis) following a large impact; see Figure 6. The evaluated methodology attempts to remove false positives based upon a dynamic threshold, estimated through an observation of the average quantified stride length (Young et al., 2020); however, the process is not consistently performant in warranting use at higher speeds. Recently, the use of deep learning has demonstrated utility in identifying temporal gait outcomes in both normal (Gadaleta et al., 2019) and running gait (Gholami et al., 2020; Johnson et al., 2020) from wearable inertial sensors but requires further investigation and validation (i.e., in a range of speeds) before adoption. Should such approaches exhibit a range of validity within the domain, their use could be warranted to inform impact-related gait feature extraction outcomes.

In contrast to gyroscope-only based methods that perform gait cycle segmentation through estimating the rotation of the foot for mid-swing analysis, the use of a ZC approach in vertical acceleration (accelerometer) for initial contact identification can help a wider understanding of running gait outcomes at the point of impact due to their sensitivity to ground forces (Falbriard et al., 2018). Crucially, by using initial contact as a marker from an accelerometer, we can search for rotation-based outcomes surrounding initial contact (e.g., pronation and foot strike location).

### Ground contact time

Ground contact time is essential to understand due to the implications for running the economy (Di Michele and Merni, 2014). The evaluated approach performs smoothly when observing mean errors from labeled data, demonstrating efficacy between 8–12 km/h (mean error 0.32–2.65%), with a degradation at 14 km/h + (3.94% mean error at 14 km/h; 10+ % mean error at self-selected); see Table 3. However, observing box and Bland Altman plots—see Figures 4, 5—one can observe that despite low deviation from average labels at higher speeds (14 km/h+), there is a significantly wider range of estimated values. These findings are comparable to similar work within the field. For example, Falbriard et al. (2018) assessed a range of signal features (e.g., min/max, ZC) for the identification of temporal gait outcomes, including ground contact time. Similar to the evaluated algorithm, utilizing optimally selected features, the approach presented a degradation in accuracy with respect to speed. However, the observed work also provides a discussion of alternate, lesser-performant features (i.e., the minimum of pitch angular velocity within the IC zone and the maximum of vertical acceleration in the TC zone) that are not significantly affected by speed, which may warrant further investigation for use in high-speed running.

### Gait feature extraction

The gait feature extraction layer of the algorithm relies upon observing the horizontal and vertical angular velocity of the foot at impact for pronation and foot strike location, respectively. Table 1 illustrates the performance of the gait feature extraction layer, demonstrating consistently excellent performance across multiple speeds for foot strike location identification, but showing degraded performance in pronation identification at higher speeds.

Upon investigating the horizontal (pronation) and vertical (foot strike location) rotational velocity planes, extraneous noise became apparent surrounding an initial contact event within the vertical rotational velocity plane at higher speeds; see Figure 6. Supplementary Table 1 shows the average noise-to-signal ratio across the range of speeds in both vertical and horizontal rotational velocity within a 167 ms (10 Hz) window of an initial contact event. The experiment demonstrates an obvious and significant increase in noise (+10.1%) in horizontal roll between slow and high speeds, explaining the degradation in pronation accuracy. Conversely, there is noticeably less noise in the vertical rotation plane at all rotational speeds, which is accompanied by consistently excellent test results. The use of a continuous wavelet transform (CWT) may be warranted in future iterations due to high performance in single-sensor applications through noise suppression by the removal of extraneous signal fluctuations, leading to clearer gait feature extractions (McCamley et al., 2012).

The proposed approach performs comparably with similar work within the field. For example, Murai et al. (2018) utilized a single, foot-mounted IMU to observe the angular velocity of the foot during impact to assess pronation at a correlation of *r* = *0.800*, coinciding with our ICC_(2,1)_ score of 0.779–0.867 between 8–12 km/h. However, our evaluated approach provides a significantly higher number of participants (31 runners of varying demographics: evaluated approach, ten male runners: observed study) and insight into the speed of the runners and how it affects performance, providing a more generalizable assessment of IMU-based pronation assessment.

### Implications of free-living running gait assessment

Through developing and evaluating an IMU-based algorithm with promising results, the approach provides scope for implementation in low-cost commercial technologies, reducing reliance on expert analysis and/or gold-standard, high-cost technologies (Young et al., 2020). The methodology investigated here primarily focuses on gait feature extraction during treadmill running for use in habitual or low-resource environments. However, there is some debate about the efficacy of treadmill-based gait assessment due to gait kinematics differing in overground and outdoor running scenarios (Lafferty et al., 2021; Benson et al., 2022), potentially inhibiting the utility of the evaluated ZC method. Consequently, the approach should be validated in outdoor scenarios to assess its performance in uncontrolled settings. In performing outdoor validation, the use of IMU-based methods could contribute to a full-scale running gait analysis, providing relatively sparse, long-term observations such as gait monitoring across 10 km or marathon running (Benson et al., 2018; Meyer et al., 2021).

### Limitations

Due to the potential for high impact forces at greater speeds, the use of a running shoe during testing was warranted to minimize the risk of impact-related injuries (Sun et al., 2020). The deployed running shoes in this study (Saucony Guide Runner) exhibit a neutral-cushioning shoe and, thus, do not provide pronation-minimizing support. However, running shoes are widely accepted to influence and change aspects of a runner's gait in opposition to barefoot running (Stacoff et al., 1991; Aguinaldo and Mahar, 2003; Jandová et al., 2018). Consequently, although the evaluated algorithms can extract pronation, foot strike location, and ground contact time in comparison to labeled data, the outcomes may not be indicative of the runner's “true” gait, i.e., when running barefoot. Additionally, running shoes reduce impact force on the lower extremities (Aguinaldo and Mahar, 2003), potentially minimizing acceleration on impact observed by the IMU in comparison to previous work (Young et al., 2020). As such, although the evaluated ZC approach to IC identification performs well within the constraints of the study, it should be noted that the ZC approach may not scale between different running shoes, i.e., those that exhibit larger levels of support.

IMUs are susceptible to drift errors due to high-frequency noise within micro-electro-mechanical systems (Narasimhappa et al., 2019) and the potential for local misalignment. Although the evaluated algorithm takes into account local alignment error and uses a Butterworth filter to account for noise, this approach may not be drift-free among different running patterns, which may impact gait outcomes. Therefore, to ensure that the approach is not hindered by drift, it may be necessary to implement a drift-minimizing algorithm (Falbriard et al., 2020). This could be especially useful in analysis at higher speeds, where sensors may be more susceptible to drift due to extensive exposure to high-impact forces.

## Future work

Currently, the evaluated algorithm degrades in performance at higher speeds (14 km/h+) due to extraneous noise encountered at higher impact speeds and misidentification of initial contact events. Some shortcomings have been identified for future iterations of the algorithm, namely requiring the use of CWT processing and the potential implementation of artificial intelligence for the identification of IC events in anomalous signals. Future work will validate the approach in overground (i.e., off-treadmill), outdoor running to assess IMU-based running gait assessment over extended running bouts.

## Conclusion

The proposed work investigates and evaluates a ZC methodology for the extraction of a range of biomechanical properties from a single foot-mounted IMU that could be useful for general running gait analysis. The evaluated method has demonstrated utility in quantifying foot strike location, pronation severity, and ground contact time during treadmill running at speeds up to 12 km/h, exhibiting good and excellent agreements with 3D motion capture. By conducting this investigation on the ZC methodology for running gait assessment, we contribute to understanding the efficacy and utility of wearable IMUs during running gait. Particularly, providing approaches to understanding running with low-cost apparatus to promote personalized and objective running gait assessment and reduce reliance on traditional, subjective approaches.

## Data availability statement

The raw data supporting the conclusions of this article will be made available by the authors, without undue reservation.

## Ethics statement

The studies involving human participants were reviewed and approved by Northumbria University Research Ethics Committee. The patients/participants provided their written informed consent to participate in this study.

## Author contributions

FY and AG conceptualized the question and hypothesis. FY, SS, and AG designed the study from which the data originates. FY and RMa contributed to data collection and analysis. FY wrote the first draft. FY, RMa, CW, RMo, SS, and AG contributed to the interpretation, writing, and editing of the manuscript. All authors contributed to the article and approved the submitted version.

## Funding

The work was supported by Northumbria University and the European Regional Development Fund (ERDF) Intensive Industrial Innovation Programme (IIIP). It was delivered through Northumbria University (Grant Number: 25R17P01847).

## Conflict of interest

The authors declare that the research was conducted in the absence of any commercial or financial relationships that could be construed as a potential conflict of interest.

## Publisher's note

All claims expressed in this article are solely those of the authors and do not necessarily represent those of their affiliated organizations, or those of the publisher, the editors and the reviewers. Any product that may be evaluated in this article, or claim that may be made by its manufacturer, is not guaranteed or endorsed by the publisher.
