# Supplementary material for: Examination of a foot mounted IMU-based methodology for a running gait assessment
Source: Front Sports Act Living. 2022 Sep 6;4:956889. doi: 10.3389/fspor.2022.956889 (PMC9485551; doi:10.3389/fspor.2022.956889)
Supplement: Supplementary file 1 [file Table_1.DOCX]

Supplementary Material

# Supplementary Tables

Supplementary Table 1: Noise-to-signal ratio of horizontal (pronation) and vertical (foot strike location) rotation signals at different speeds. The following table illustrates how, at higher speeds there is considerably higher rates of noise within the signal. As such, the distortion within the IMU acceleration may affect overall results of the evaluated algorithms.

| **Speed (km/h)** | **8** | **10** | **12** | **14** | **Self Selected** |
| --- | --- | --- | --- | --- | --- |
| Horizontal Roll Signal Noise | 0.038 | 0.049 | 0.068 | 0.093 | 0.139 |
| Vertical Roll Signal Noise | 0.0028 | 0.0087 | 0.0045 | 0.0082 | 0.0073 |

# Supplementary Figures
